# Flow Cytometric Aldehyde Dehydrogenase Assay Enables a Fast and Accurate Human Umbilical Cord Blood Hematopoietic Stem Cell Assessment

**DOI:** 10.4274/tjh.2016.0214

**Published:** 2017-12-01

**Authors:** Emine Begüm Gençer, Pınar Yurdakul, Klara Dalva, Meral Beksaç

**Affiliations:** 1 Ankara University Faculty of Medicine, Cord Blood Bank, Ankara, Turkey; 2 Ankara University Faculty of Medicine, Biotechnology Institute, Ankara, Turkey; 3 TOBB Economics Technology and University Faculty of Medicine, Department of Medical Microbiology, Ankara, Turkey; 4 Ankara University Faculty of Medicine, Stem Cell Research Institute, Ankara, Turkey; 5 Ankara University Faculty of Medicine, Department of Hematology, Ankara, Turkey

**Keywords:** Cord blood, Aldehyde dehydrogenase, Colony-forming unit-granulocyte/macrophage

## Abstract

**Objective:**

Colony-forming units of granulocytes/macrophages (CFU-GM) analysis is the most widely used method to determine the hematopoietic stem cell (HSC) content of human umbilical cord blood (CB) for prediction of engraftment potential. The measurement of aldehyde dehydrogenase (ALDH) activity is a more recent method for HSC qualification. Our aim was to correlate phenotypic and functional assays to find the most predictive method.

**Materials and Methods:**

In this study, flow cytometric quantitation of CD34^+^ cells and ALDH positivity along with CFU-GM capacity were assessed in fresh and post-thaw CB units.

**Results:**

Among 30 post-processing samples, for each CB unit the mean total number of nucleated cells (TNCs) was (93.8±30.1)x10^7^, CD34^+^ cells were (3.85±2.55)x10^6^, ALDH^+^ cells were (3.14±2.55)x10^6^, and CFU-GM count was (2.64±1.96)x10^5^. Among an additional 19 post-thaw samples the cell counts were as follows: TNCs, (32.79±17.27)x10^7^; CD34^+^, (2.18±3.17)x10^6^; ALDH^+^, (2.01±2.81)x10^6^; CFU-GM, (0.74±0.92)x10^5^. Our findings showed that in fresh samples TNCs, CD34^+^ cells, and ALDH correlated highly with counts of CFU-GM, CFU-erythroids/granulocytes-macrophages/megakaryocytic cells (GEMM), and burst forming units of erythroids (BFU-E) as follows: TNCs, r=0.47, r=0.35, r=0.41; CD34+, r=0.44, r=0.54, r=0.41; and ALDH, r=0.63, r=0.45, r=0.6, respectively. In terms of post-thaw samples, the correlations were as follows: TNCs, r=0.59, r=0.46, r=0.56; CD34^+^, r=0.67, r=0.48, r=0.61; and ALDH, r=0.61, r=0.67, r=0.67, for CFU-GM, CFU-GEMM, and BFU-E, respectively. All correlations were statistically significant.

**Conclusion:**

In our experience, HSC assessment by ALDH activity yields the highest correlation with conventional analytical methods, particularly for post-thaw samples. Thus, this fast, inexpensive method has the potential to overcome the weaknesses of other techniques.

## Abstract (tr)

**Amaç:**

Granülositer makrofaj koloni oluşturma (CFU-GM) testi kordon kanı (KK) hematopoietik kök hücre engrafman potensiyelini ölçmek için kullanılan bir yöntemdir. Aldehit dehidrogenaz (ALDH) enzimi ölçüm yöntemide hematopoetik kök hücre (HKH) kalitesini belirlemek amacıyla kullanılan daha yeni bir metottur. Çalışmamızda fenotipik ve fonksiyonel olarak korelasyon analizi yapılarak HKH ölçümünde en etkili metodu bulmayı amaçladık.

**Gereç ve Yöntemler:**

Bu çalışmada taze ve donma çözme sonrası KK ünitelerinde CD34+ ve ALDH+ hücrelerle CFU-GM kapasiteleri araştırılmıştır.

**Bulgular:**

NOtuz taze KK ünitesinde her KK için ortalama değerler: Toplam çekirdekli hücre sayısı (TNC): 93,8±30,1x107, CD34+: 3,85±2,55x106, ALDH+: 3,14±2,55 x106, CFU-GM: 2,64±1,96x105. On dokuz KK ünitesinde donma çözme sonrası hücre değerleri: TNC: 32,79±17,27x107, CD34+: 2,18±3,17x106, ALDH+: 2,01±2,81x106, CFU-GM: 0,74±0,92x105’dir. Bulgularımız; taze KK’da TNC, CD34 ve ALDH; CFU-GM, CFU-GEMM ve BFU-E ile korelasyon gösterirken (TNC, r=0,47, r=0,35, r=0,41; CD34+, r=0,44, r=0,54 r=0,41; ve ALDH, r=0,63 r=0,45 r=0,6) donma çözme sonrası KK’da korelasyon sırasıyla CFU-GM, CFU-GEMM, ve BFU-E için, TNC r=0,59, r=0,46, r=0,56, CD34+ r=0,67, r=0,48, r=0,61 ve ALDH r=0,61, r=0,67, r=0,67 olarak saptanmıştır. Bütün bulgularımız istatistiksel olarak anlamlı çıkmıştır.

**Sonuç:**

Çalışmamız, ALDH aktivitesi tayin metodu HKH tayininde geleneksel yöntemlerle özellikle donma çözme sonrası örnekler açısından korelasyon göstermiştir. Böylelikle hızlı, ucuz bir metod olarak ALDH diğer HKH belirlemede kullanılan yöntemlere üstün olabilecek kapasitededir.

## INTRODUCTION

Recent scientific evidence demonstrates that different subtypes of CD34^+^ cells in the cord blood (CB) hematopoietic stem cell (HSC) niche have different engraftment potentials [1,2]. It is of crucial importance to determine the quality of the CB particularly following freeze/thaw cycles. Two different approaches can be used to assess the functionality and population-forming capacities of CB HSCs along with the gold standard method of the International Society of Hematotherapy and Graft Engineering (ISHAGE) [3]. Ex vivo colony-forming unit (CFU) assays are the most widely used tests for determining HSC functions, but they possess serious drawbacks such as difficulty in routine application, lack of standardization, labor-intensive nature, and long turnaround time [4]. One of the likely reasons for this is probably the fact that while being predictive of short-term re-populating cells, CFU assays could not determine long-term populating cells effectively. Long-term populating cells have been shown to provide long-term immune reconstitution after CB transplantation (CBT); thus, it is of crucial importance to assess their numbers. The measurement of aldehyde dehydrogenase (ALDH) activity can therefore be much more accurate due to the intracellular presence of this enzyme [5].

It was reported that ALDH enzyme expression is high in early HSCs in the bone marrow and CB [6,7]. A few published studied correlated high ALDH activity with better permanent engraftment following HSC transplantation [5,7,8,9,10,11]. In the first such study by Lioznov et al. [12], it was reported that ALDH expression is a practical marker to assess HSC activity for both stem and progenitor cells before bone marrow and peripheral blood transplantation. There are hardly any data for CB investigating the phenotypic and functional properties of CB HSCs and the correlation of ALDH activity with CFU potential in pre- and post-thaw CB HSCs [5,7,11,13,14]. In this study, we aimed to correlate phenotypic assays with functional assays to find the most predictive method for fresh and post-thaw CB.

## MATERIALS AND METHODS

### CB Unit Selection and Processing

A total of 50 CB units from consenting maternal donors collected at the Ankara University Faculty of Medicine’s Cord Blood Bank were included in this study. Thirty CB units that met volume and total number of nucleated cell (TNC) eligibility criteria (>70 mL and 100x10^7^/U, respectively) were processed and used immediately for the fresh group and 20 non-conforming CB units that had been reserved for research purposes were included as the post-thaw group (1 unit was discarded due to CFU culture contamination). CB units were processed automatically with a Sepax 2 device (Biosafe) and TNC counts, post-processing CD34^+^ cell enumeration, and cell viabilities were assessed for every CB unit.

### Post-Thaw Washing

Nineteen non-conforming CB units were thawed in a 37 °C water bath and samples of 10 mL were taken into conical tubes. In order to remove DMSO, CB units were washed using a washing solution (10% dextran 40 (BioFleks), 20% human serum albumin (Centurion Pharma), and PBS (Lonza) at 4:1:3/8 (v/v), respectively). Upon thawing of the CB units they were washed twice with 1:1 (v/v) washing solution After discarding the supernatant, the pellet was re-suspended in washing solution by gentle mixing.

### Determination of TNC/CD34 Viability and Counts

The number of TNCs for all units (30 fresh and 19 post-thaw units) was assessed by complete blood counting with an automated cell counter (Beckman Coulter, LH780). CB unit CD34^+^ cell enumeration and detection of cell viability by 7-aminoactinomycin dye was performed using a Stem-Kit upon the acquisition of the data with an FC 500 instrument (Beckman Coulter). The analysis was performed using an ISHAGE single test platform.

### ALDH Analysis

The ALDEFLUOR assay (StemCell Technologies) was used for the detection of ALDH expression in fresh and post-thaw CB HSCs. ALDH activity was measured by the protocol recommended by the manufacturer. Briefly, cell suspensions were adjusted to 10^6^ cells/mL with 1500 µL of ALDEFLUOR assay buffer after red blood cell depletion. ALDEFLUOR reagent (10 µL) was added to each tube, followed by 5 min of centrifugation at 300×g. Supernatants were stained with FITC-ALDH, APC A-750-CD38, phycocyanin (PC) 7-CD34, chrome orange-CD45 (Beckman Coulter), PE-CD73, and PC 5-CD90 (Becton Dickinson) antibodies and analyzed by flow cytometry (Beckman Coulter FC500). Diethylaminobenzaldehyde reagent was used to suppress ALDH activity in control tubes. Using the ALDH activity assay, CB HSCs were categorized as ALDH^+^ and ALDH^-^.

### CFU Assays

CFU assays were implemented according to the manufacturer’s recommendations (StemCell Technologies CFU Manual, MA28404) and modified from Lee et al. [19]. First, 100 µL of CB sample was removed from all CB units, and after the addition of 80 µL of HetaSep and 300 µL of Iscove’s modified Dulbecco medium containing 2% fetal bovine serum (StemCell Technologies), the mixture was incubated at 37 °C in 5% CO_2_ for 20 min (Sanyo CO_2_ Incubator). Cells (5x10^5^ cells/mL) were transferred to 3 mL of MethoCult Express medium. After 14 days, colonies were counted and different morphologies as well as numbers of CFUs were recorded using an inverted light microscope (Olympus/IX51). The number of colonies was calculated as the mean value for two dishes.

### Statistical Analysis

CFU assays were implemented according to the manufacturer’s recommendations (StemCell Technologies CFU Manual, MA28404) and modified from Lee et al. [19]. First, 100 µL of CB sample was removed from all CB units, and after the addition of 80 µL of HetaSep and 300 µL of Iscove’s modified Dulbecco medium containing 2% fetal bovine serum (StemCell Technologies), the mixture was incubated at 37 °C in 5% CO_2_ for 20 min (Sanyo CO_2_ Incubator). Cells (5x10^5^ cells/mL) were transferred to 3 mL of MethoCult Express medium. After 14 days, colonies were counted and different morphologies as well as numbers of CFUs were recorded using an inverted light microscope (Olympus/IX51). The number of colonies was calculated as the mean value for two dishes.

## RESULTS

In this study we aimed to compare three different methods in terms of efficiency to assess different re-populating HSCs from CB, both after processing and after thawing. We analyzed different cellular fractions, namely TNCs; CD34^+^, ALDH^+^, CD34^+^ ALDH^+^, ALDH^+^ CD34^+^, and ALDH^+^ CD34^+^ CD90^+^ CD38^-^ cells; and colony-forming units of granulocytes/macrophages (CFU-GM), CFU-erythroids/granulocytes-macrophages/megakaryocytic cells (GEMM), and burst forming units of erythroids (BFU-E), for both fresh and post-thawed units. Table 1 demonstrates the mean, median, and minimum-maximum values of the aforementioned parameters for fresh and post-thawed samples.

Table 2 provides the correlation values for ALDH positivity and TNC, CD34^+^, and CD34^+^ CD90^+^ CD38^-^ cell numbers as well as CFU-GM, CFU-GEMM, and BFU-E colony counts among all CB samples. When fresh samples were analyzed, ALDH activity correlated well with all the cell populations investigated; TNC, ALDH^+^, and CD34^+^ fractions were found to be highly correlated both with CFU-GM, CFU-GEMM, and BFU-E and with each other. The correlation coefficients remained significant for fresh and post-thawed samples, and when post-thaw data were analyzed, TNCs, CD34+, and ALDH were also found to be statistically correlated with CFU-GM, CFU-GEMM, and BFU-E (Table 2).

**Figure 1:**
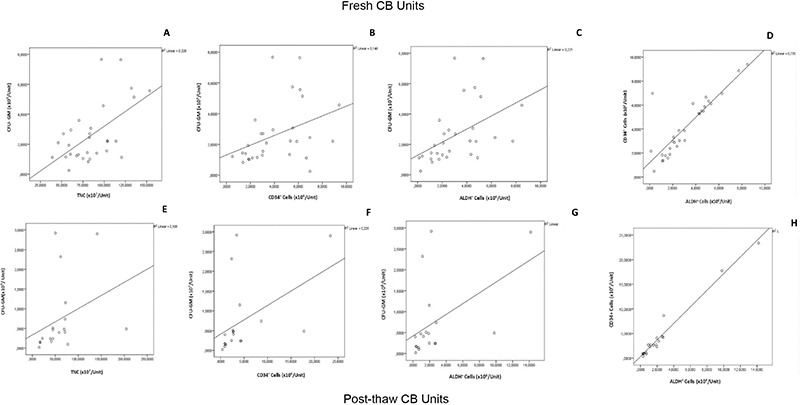
Correlation values of TNCs, ALDH, and CD34^+^ cells with CFU-GM and ALDH^+^ CD34^+^ cells. Graphs A-D denote correlations for fresh CB units: TNCs and CFU-GM (A); CD34^+^ cells and CFU-GM (B); ALDH^+^ cells and CFU-GM (C); CD34^+^ cells and ALDH^+^ cells (D). Graphs E-H denote correlations for post-thaw samples: TNCs and CFU-GM (E); CD34^+^ and CFU-GM (F); ALDH^+^ and CFU-GM (G); CD34^+^ cells and ALDH^+^ cells (H).

Among all parameters compared, the most striking correlation was detected for CFU-GM numbers and ALDH positivity for fresh CB units (r=0.629, p<0.001); post-thaw analyses also revealed a correlation for CFU-GM and ALDH^+^ cells when the same parameters were investigated (r=0.608, p=0.006; Table 2). When CFU-GM numbers were tested against all parameters for post-thawed samples, an even higher correlation was detected in CD34^+^ cells, which were also positive for ALDH (r=0.670, p=0.002). On the other hand, there was no significant correlation between TNCs and ALDH^+^ cells, which were also positive for CD34 (r=0.432, p=0.065). These correlations are shown in Figure 1. Table 2 shows r values for all parameters investigated.

## DISCUSSION

One of the recent methods described for the rapid and accurate detection of functional CB cell fractions is ALDH activity measurement in CB HSCs. To date, five studies have looked at ALDH levels in CB units [5,7,11,13,14]. Our study is unique in terms of having a detailed post-thaw analysis and to our knowledge it is also the first to determine the capacity of ALDH^+^ CD90^+^ CD34^+^ CD38^-^ cells, a group of cells that possess high engraftment capacity. Most of the papers in the literature have focused on HSCs with high ALDH activity with conflicting results related to their role in engraftment following HSC transplantation [11,14,15,16]. An experiment carried out by Pearce et al. [17] showed that ALDH and CD34 double-positive cells constitute 63% of lineage-negative cells for TNCs and only ALDH^+^ cells improved engraftment. Storms et al. [18] classified HSCs as being CD34^+/-^, ALDH^+^, and ALDH^-^ and demonstrated that only CD34^+^ ALDH^+^ cells were efficient in terms of long-term and short-term re-population capacities.

Shoulars et al. [11] developed an ALDH-based method to estimate the post-thaw quality of CB units. The results of their study, similar to ours, demonstrated that ALDH activity is highly correlated with CFU counts and can be integrated into routine CB unit release procedures prior to transplantation. Thus, our findings, which show the highest correlation between in vitro CFU counts and ALDH activity compared to TNCs or CD34, are confirmed by this very recent publication.

We aimed to compare three different approaches to assess different re-populating HSCs from CB, both fresh and after thawing. In our study, CD34^+^ cells were found to constitute 0.49±0.26% of all TNCs and 0.35±0.21% of ALDH^+^ cells. Additionally, 86.98±13% of all CD34^+^ cells were found to be ALDH^+^, and within all ALDH^+^ cells, 94.54±5.3% were CD34^+^. Among all CB units tested, the rate of ALDH^+^ CD34^-^ cells was found to be 5.46% and 13.02% were ALDH^-^ but CD34^+^. Different research groups sought to identify different cellular populations by ALDH staining intensities, but only two of them compared the ALDH activity of different CB sub-populations [5,19]. The clinical significance of those populations remains to be determined. In a study by Lee et al., [7] CD34 cells were found to constitute 0.14±0.10% of all TNCs. In comparison, CD34 positivity was seen in 0.49±0.26% of all TNCs in our study. Unlike our results, Lee et al. [7] detected less ALDH positivity among CD34^+^ cells (74.5±13.8%), and of the entire ALDH^+^ population, 69.9±15.5% of cells were shown to express CD34. Another similar investigation by Storms et al. [18] demonstrated even higher HSC rates in fresh CB samples: 0.9±0.5% of TNCs were CD34^+^, but 47.9±14.3% of those cells were ALDH^+^. ALDH^+^ cells constituted 0.96±0.5% of TNCs and 50.9±18.3% of ALDH+ cells were CD34+ [18]. In Gentry et al.’s [10] study CD34^+^ cell count was found to be 0.15±0.08% of all TNCs, and 0.05±0.02% of TNCs expressed high ALDH.

Attia et al. [14] reported that ALDH activity detection is not only quick and easy to perform but also it does not affect the cell viability or re-populating capacity of CB cells, which may be a serious drawback of some CD34 detection systems [20]. Ikeda et al. [13] suggested that prior to CBT the ALDH assessment method could be an alternative approach to the selection of CB units for unrelated donors [14]. All of the results from the papers mentioned here are in favor of our findings indicating the utility of an ALDH-based approach for CBT settings.

Characterization of the sub-populations of CB is crucial because high cell doses with adequate viability predict the outcome after CBT. Engraftment is generally ensured when highly CD34^+^ cells are used, but occasionally a graft with partially dysfunctional cells due to freeze/thaw processes can affect the cells’ short-term and long-term re-populating capacities [20]. In vitro manipulations have been shown to interfere with membrane CD34 expression without hampering HSC functionality [21,22]. With the ALDH analysis approach, HSCs with relatively high engraftment capacity but with limited or no growth in CFU tests can easily be detected.

As we sought to determine the correlation levels for TNCs and ALDH^+^ and CD34^+^ cells with CFU capacities for both fresh and post-thaw samples, we demonstrated that ALDH positivity correlated highly with CFU-GM capacity (r=0.629, p<0.001) in fresh samples. On the other hand, the highest correlation was detected for CFU-GM numbers and CD34^+^ cells for the post-thaw group of CB units (r=0.655, p<0.001) (Table 2). With a similar approach, Lee et al. [19] analyzed ALDH^+^, CD34^+^, ALDH^+^ within CD34^+^, and CD34^+^ within ALDH^+^ cell populations and CFU-GM and CFU-GEMM capacities in 245 CB units, both fresh and after thawing. Unlike our results, CFU-GM count was not found to be correlated with TNCs in their study. In addition to Lee et al.’s [19] approach, ALDH^+^ CD90^+^ CD34^+^ CD38- cell populations and BFU-E capacities were also analyzed in our study. Lee et al. [19] did not provide any data related to post-thaw samples in terms of TNC/ALDH and TNC/CD34+ ratios, but we demonstrated in our study that 0.66±0.4% of TNCs were CD34^+^ cells and 0.37±0.27% of TNCs were ALDH^+^ cells.

In a phase 1 study by Gentry et al. [10], CD34^+^ cell counts were claimed to be the sole post-thaw CB quality predictor, indicating a lower transplant-related mortality, but post-thaw comparisons of CFU-GM counts versus ALDH activity were not conducted. In our study, ALDH^+^ cells correlated well with CFU-GM for post-thaw samples (Table 2). Similar to our results, a positive correlation was detected between ALDH^+^ cells and CFU-GM positivity (r=0.40, p=0.03) in Frandberg et al.’s [5] study, but their work did not reveal any correlation of CD34^+^ cells with total CFU count (r=0.36, p=0.051).

In addition to ALDH positivity, determination of CD90^+^ CD34^+^ CD38^-^ cells may also be a good predictor of engraftment both for fresh and post-thaw CB units. The ALDH^+^ CD90^+^ CD34^+^ CD38^-^ group of progenitors is unique with their high engraftment and re-populating capacities [23,24,25]. To our knowledge, our study is the first to examine the ALDH capacity of these particular cells. When we analyzed ALDH positivity in CD90^+^ CD34^+^ CD38^-^ cells, CFU-GM, CFU-GEMM, and BFU-E counts were found to be well correlated for both fresh and post-thaw CB units (Table 2). Putman et al. [9] performed CFU tests for ALDHhi and ALDHlo cellular populations of CB BFU-E, CFU-GM, and CFU-GEMM. In our study the highest correlation of ALDH+ cells was found with CFU-GM and BFU-E for fresh samples. Putman et al. [9] found that the CB ALDHhi population was significantly enriched for human hematopoietic progenitor function.

Owing to the nature of ALDH as an intracellular enzyme, it may be less affected by centrifuge force and thus may reflect the actual HSC population in a more realistic manner. By using ALDH as a marker of functionality, the disadvantage of a likely false negativity caused by CD34^+^ cell counting could also be overcome. Similar to our results, Shoulars et al. [11] from Duke University recently demonstrated that ALDH activity measured from post-thaw segments highly correlated with CFUs. They suggested that measurement of ALDHbr CD34^+^ cells might indicate CFU potency and thus engraftment capacity.

## CONCLUSION

In light of our results and other recently published studies, we propose that ALDH activity determination can substitute for CFU-GM tests. This fast and inexpensive method has the potential to overcome the weaknesses of other techniques, such as the limitations of CD34 counting due to the internalization of membrane CD34 expression or lack of standardization and long turnaround time of CFU assays. We are aware of the limitations in engraftment prediction by phenotype-based analysis. ALDH measurement, as confirmed by us, has the highest correlation with in vitro functional assays. Currently CD34 and CFU-GM assays, accepted as golden standards, are expected to be replaced by ALDH measurement, which is a fast, reproducible, and accurate assessment tool.

## Figures and Tables

**Table 1:**
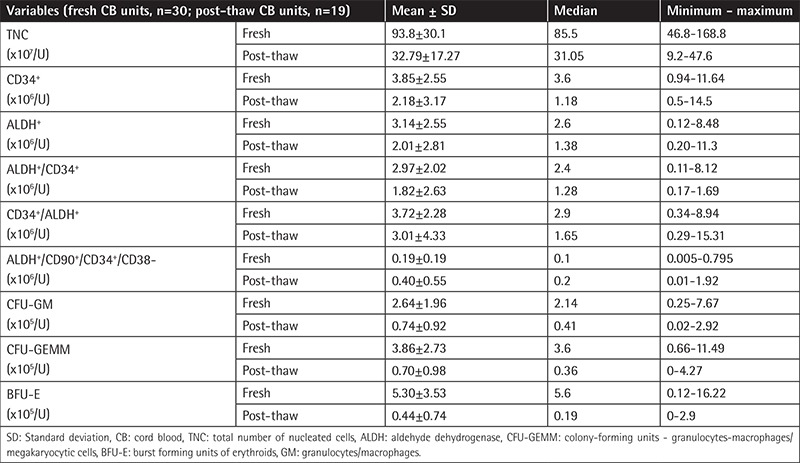
Characteristics of all fresh and post-thaw CB units tested.

**Table 2:**
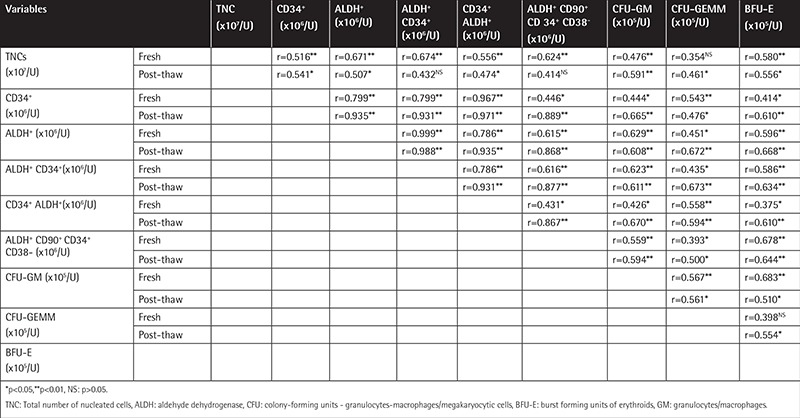
Correlations between different groups of cells demonstrating hematopoietic activity in fresh and post-thaw cord blood units.
